# Validation of a Sensor-Based Gait Analysis System with a Gold-Standard Motion Capture System in Patients with Parkinson’s Disease

**DOI:** 10.3390/s21227680

**Published:** 2021-11-18

**Authors:** Verena Jakob, Arne Küderle, Felix Kluge, Jochen Klucken, Bjoern M. Eskofier, Jürgen Winkler, Martin Winterholler, Heiko Gassner

**Affiliations:** 1 Movement and Gait Lab, Sana-Krankenhaus Rummelsberg, 90592 Schwarzenbruck, Germany; verena.jakob@sana.de; 2 Machine Learning and Data Analytics Lab, Department Artificial Intelligence in Biomedical Engineering (AIBE), Friedrich-Alexander-Universität Erlangen-Nürnberg (FAU), 91052 Erlangen, Germany; arne.kuederle@fau.de (A.K.); felix.kluge@fau.de (F.K.); bjoern.eskofier@fau.de (B.M.E.); 3 Digital Medicine, University of Luxembourg, 4365 Esch-sur-Alzette, Luxembourg; jochen.klucken@uni.lu; 4 Digital Medicine, Luxembourg Institute of Health, 1445 Strassen, Luxembourg; 5 Department of Molecular Neurology, University Hospital Erlangen, Friedrich-Alexander Universität Erlangen-Nürnberg (FAU), 91054 Erlangen, Germany; juergen.winkler@uk-erlangen.de; 6 Department of Neurology, Sana-Krankenhaus Rummelsberg, 90592 Schwarzenbruck, Germany; martin.winterholler@sana.de; 7 Fraunhofer IIS, Fraunhofer Institute for Integrated Circuits IIS, 91058 Erlangen, Germany

**Keywords:** Parkinson’s disease, wearables, inertial sensors, three-dimensional gait analysis, machine learning algorithm, spatiotemporal gait parameters

## Abstract

Digital technologies provide the opportunity to analyze gait patterns in patients with Parkinson’s Disease using wearable sensors in clinical settings and a home environment. Confirming the technical validity of inertial sensors with a 3D motion capture system is a necessary step for the clinical application of sensor-based gait analysis. Therefore, the objective of this study was to compare gait parameters measured by a mobile sensor-based gait analysis system and a motion capture system as the gold standard. Gait parameters of 37 patients were compared between both systems after performing a standardized 5 × 10 m walking test by reliability analysis using intra-class correlation and Bland–Altman plots. Additionally, gait parameters of an age-matched healthy control group (*n* = 14) were compared to the Parkinson cohort. Gait parameters representing bradykinesia and short steps showed excellent reliability (ICC > 0.96). Shuffling gait parameters reached ICC > 0.82. In a stridewise synchronization, no differences were observed for gait speed, stride length, stride time, relative stance and swing time (*p* > 0.05). In contrast, heel strike, toe off and toe clearance significantly differed between both systems (*p* < 0.01). Both gait analysis systems distinguish Parkinson patients from controls. Our results indicate that wearable sensors generate valid gait parameters compared to the motion capture system and can consequently be used for clinically relevant gait recordings in flexible environments.

## 1. Introduction

Parkinson’s disease (PD) is currently the world’s fastest-growing neurological disorder and characterized by motor and non-motor symptoms that worsen with disease progression [1]. The main symptoms are the presentation of bradykinesia, rigidity, tremor and postural instability [1]. Gait impairment in PD is often characterized by short steps and a shuffling gait resulting in an increased risk of falling [2]. Gait impairment plays an important role for PD patients, as well as affects the quality of life, limits the independence, and reduces activities of daily living [3]. To determine the severity of gait disorders, an early and objective gait assessment is important [4,5].

Motion-capture (MC)-based gait analysis is still the gold standard to provide a parameter for kinematics and kinetics of human gait and, thus, provides an ideal confirmation measure for inertial sensor-based gait analysis [5,6,7]. Passive reflective markers are placed on the lower extremities, pelvis and trunk of the participants to standardized protocols [5]. Special high-frequency cameras calculate trajectories of these markers and produce quantified, reliable, and accurate results over short-distance walking tests. As data acquisition is time consuming, expensive, and can be performed by specialized personnel solely, a three-dimensional gait analysis using motion capture systems still requires enormous effort and is rarely practicable to use in a home-monitoring setting [4]. 

For a quick and mobile assessment of gait, wearable sensors provide an alternative measurement technique [8]. Mobile systems have the potential to record gait patterns over several hours in flexible environments [9,10]. Body-worn inertial sensors comprising of biosensors such as accelerometers and gyroscopes, combined with signal processing and machine learning algorithms, measure changes of gait patterns objectively and by metric [3,10,11,12]. Moreover, it has already been shown that a sensor-based gait analysis system was able to distinguish PD patients from controls and allowed automated staging and symptom monitoring in PD [10]. Previous studies already showed that inertial sensors complement the clinical workup by measuring clinically relevant data, such as motor symptoms, risk of falling, or freezing of gait, that cannot be observed by eye, not only in clinical settings but also in the home environment [3,10]. In the literature, already one study with a limited number of PD patients and a healthy cohort exists, in which the validity of a sensor-based gait analysis system were compared with an MC [13].

Therefore, the aim of the study was to technically validate a mobile sensor-based gait analysis system (MGL) by comparing gait parameters with a gold-standard MC in a larger PD cohort. The hypothesis of this study was that sensor-based gait parameters describing Parkinsonian gait are reliable in comparison to gait parameters measured by an MC. For clinical validation, PD gait parameters of both systems were compared with those of an age-matched healthy control group. 

## 2. Materials and Methods

### 2.1. Study Cohort

Thirty-seven patients diagnosed with sporadic PD were recruited at the neurological clinic, Sana-Hospital Rummelsberg, Schwarzenbruck, Germany, between April 2018 and March 2019. PD patients enrolled for this study were part of the inpatient “Parkinson’s Disease Multidisciplinary Complex Treatment” program in which pharmacological treatment is combined with physiotherapy, occupational therapy, speech and language therapy, specialized balance and gait training and a cognitive behavioral training [14,15,16]. Sporadic PD was defined according to the Guidelines of the German Association for Neurology (DGN), similar to the UK PD Society Brain Bank criteria [17]. Exclusion criteria were cognitive impairment as defined by a Minimal Mental Status (MMST) < 26, as well as motor fluctuations, dyskinesia, and comorbidities potentially affecting gait (e.g., polyneuropathy, hydrocephalus, visual problems orthopedic or psychiatric comorbidities). PD patients with age > 18 years and Hoehn and Yahr disease stage (H&Y) between I–IV were either able to walk independently or safe with a walking aid (wheeled walker) without external help of another person. The gait analysis was performed in stable ON medication without the presence of clinically relevant motor fluctuations during the assessment [18,19]. A control group consisting of 14 age-matched healthy participants without any neurological or orthopedic comorbidities was also involved in the study.

Characteristics of the study population are presented in Table 1. This study was approved by the local ethics committee (reference number: 166_18 B Medical Faculty, Friedrich-Alexander-Universität Erlangen-Nürnberg, Germany), and the participants gave written informed consent according to the Declaration of Helsinki.

### 2.2. Study Procedure

**Figure 1:**
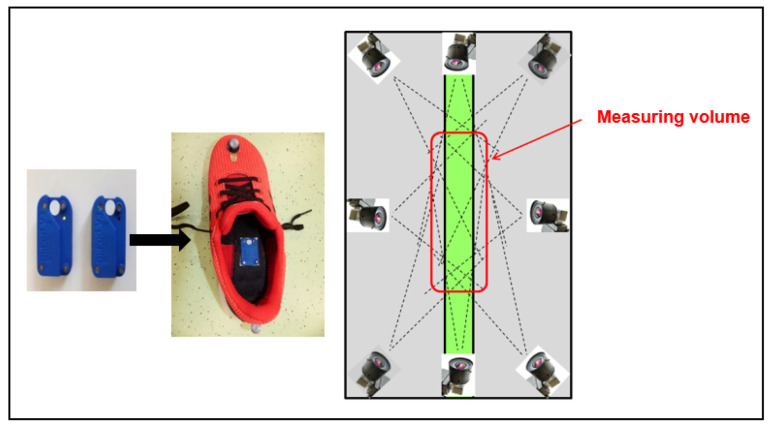
Setup: Inertial sensors and athletic shoes (**left**); measurement volume limited through cameras of MC (**right**).

PD patients performed a standardized 5 × 10 m walking test with self-selected velocity using marker-based MC (Simi Reality Motion Systems GmbH: Unterschleißheim, Germany) consisting of eight high-speed cameras (mvBlueCOUGAR Matrix Vision GmbH: Oppenweiler, Germany with 2.0MP@100Hz). In parallel, MGL (Portabiles-HCT GaitLab-System; Portabiles HealthCare Technologies GmbH: Erlangen, Germany) was used consisting of inertial sensors (miPod 3 sensors, gyroscopes and accelerometers). The sensors were integrated in the mid-sole of the athletic shoes (Figure 1). Recordings were performed using an (tri-axial) accelerometer range of ±6 g (sensitivity 300 mV/g), a gyroscope range of ±500°/s (sensitivity 2 mV/°/s), and a sampling rate of 102.4 Hz. Sensor signals were streamed via Bluetooth^®^ to a tablet computer and stored for subsequent data analysis [10,18,20]. Inertial sensor data were processed with a pattern recognition algorithm for computing clinically relevant spatiotemporal gait parameters (gait velocity, stride length, stride time, relative stance time, relative swing time, heel strike (HS) angle, toe off (TO) angle, and maximum foot clearance (TC)). The algorithm used for MGL is validated for elderly [19], young controls [21], and PD patients [13]. Additionally, passive reflecting markers were fixed based on standardized protocols on the patients’ shoes to calculate the gait parameters with the MC [22,23]. Gait velocity, stride length, stride time, relative stance time and relative swing time are used to describe the PD-related symptoms bradykinesia and short steps; the parameters HS, TO and TC represent shuffling of gait. HS angle is defined as the angle between the foot and the floor at initial foot contact (beginning of the stance phase). TO angle is defined as the angle between the foot and the ground during push off at the end of the stance phase [12,20,24]. Only straight strides were automatically detected and used for gait parameter calculations as described [19]. The MGL records the patients’ strides over the total walkway; the arrangement of the cameras limits the measurement volume of the MC to 3 × 6 m. Due to the different disease stages of the patients, only three left and three right steps were included for final analysis. For this reason, solely these steps were additionally calculated for the MGL (MGL-MV) in order to compare the gait parameters with the MC in the measurement volume (MV).

### 2.3. Statistical Analysis

Normal distribution of data was tested by the Kolmogorov–Smirnov test, and the variance homogeneity was assessed by the Levene test. As the gait parameters were partly not normally distributed, non-parametric analysis was performed for all gait parameters to compare the results between both measurement systems. Reliability analysis was performed using inter-class correlation coefficient (ICC). The mean values of the gait parameters of both systems were compared by the Wilcoxon rank test. Due to the fact that MC consists of an image-based camera-technique, 786 left and 783 right strides of 33 patients could be synchronized via video image between MC and MGL and compared by Bland–Altman plots. The Mann–Whitney U test was used to compare the gait parameters of PD patients with those of the control group. In order to minimize the effect of multiple comparisons, the significance level was adapted with the False-Rate-Discovery test by Benjamini–Hochberg for multiple testing. Values with *p* < 0.05 after the Benjamini–Hochberg correction were considered as significant. For interpretation of the ICCs, the suggestions of Koo and Li who defined values greater than 0.90 as having an excellent reliability were used [25]. For the interpretation of the Spearman’s rank correlation, values larger than 0.70 were defined as having strong correlation whereby a correlation of rho_s_ = 1.00 describes a perfect association. All statistical analyses were performed using SPSS software package version 25 (IBM Corp. Released 2017. IBM^®^ SPSS^®^ Statistics for Windows, Armonk, NY, USA: IBM Corp.).

## 3. Results

### 3.1. Correlation of MGL and MC Gait Parameters

The gait parameter reflecting bradykinesia and short steps measured by MGL, MGL-MV, and MC showed an excellent reliability as defined by ICC value > 0.90 (Table 2). The range between minimum and maximum of the 95% confidence interval presented an excellent reliability as well. ICCs of the shuffling gait parameters were lower (≥0.822), whereby the minimum of 95% confidence interval identified low to moderate reliability.

Gait parameters representing bradykinesia and short steps, analyzed with MGL and MC, did not differ significantly except for the stride time (*p* = 0.003) between systems, whereas the shuffling gait parameters differed significantly between both gait analysis systems (*p* < 0.001; Table 3). 

**Figure 2:**
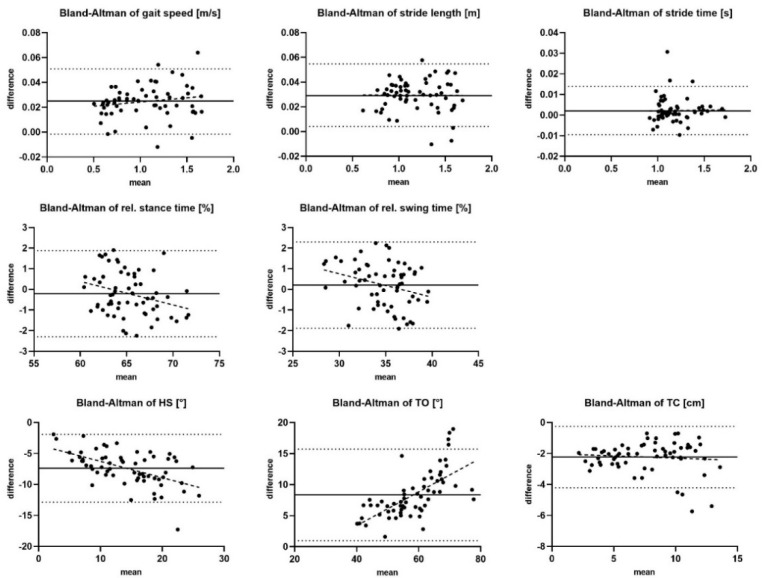
Difference between MGL and MC (bias = bold line) and ±1.96 std (dotted line) presented with Bland–Altman plots of all measured gait parameters.

From the synchronized 786 left and 783 right strides of 33 patients, it is seen that 95% of all values of gait parameters (except TO and TC) were within ±1.96 standard deviation of the mean difference—limits of agreement (Figure 2). Additionally, the Bland–Altman plots showed a constant bias between both the gait analysis system for gait speed (0.02 ± 0.01 m/s; *p* < 0.001) and the stride length (0.03 ± 0.01 m; *p* < 0.001), whereby the MGL showed larger values. For relative stance and swing time, a negative trend with higher mean values was observed (*p* = 0.198). The difference of HS (*p* < 0.001) and TO (*p* < 0.001) between the gait analysis systems increased with higher mean values. The TC also showed a constant bias of 2.25 ± 1.01 cm (*p* < 0.001) between both systems, whereby the MGL underestimated the TC. 

### 3.2. Clinical Relevance of Gait Analysis Systems—Comparison with Control Group

After correction of the significance level with the Benjamini–Hochberg false-rate-discovery test, MGL and MC measured significantly different gait parameters between patient and control group. Post hoc analysis showed that all gait parameters, except rel. stance and rel. swing phase, were significantly distinguished between patients with H&Y 3 and the control group with both gait analysis systems comparable. Higher disease staging (H&Y 4) led to strong differences in all gait parameters. In contrast, both systems did not detect gait parameter differences between patients staged with H&Y 1 + 2 and controls (Table 4).

## 4. Discussion

The aim of the study was to validate the MGL against an MC. The main findings of the study were that sensor-based gait parameters representing bradykinesia and short steps of patients with PD presented excellent reliability in comparison to MC. Additionally, both systems similarly measure significant differences between the Parkinsonian gait compared with gait patterns of an age-matched control group. Following these results, the hypothesis of this study can be confirmed.

### 4.1. Sensor-Based Gait Analysis System Provides Valid Results in PD Patients

The results of our validation study showed a strong correlation between the mobile sensor-based and MC gait analysis system. Kluge et al. validated the previous version of this sensor-based gait analysis system with an image-based MC in PD patients [13]. Compared to this study, they used silhouette tracking instead of passive reflecting markers for calculating the trajectories and analyzed solely bradykinesia and short-step parameters in a smaller cohort of PD patients and healthy probands. The results suggested that both systems can measure gait parameters comparably with good test–retest reliability in eleven analyzed healthy participants; however, the four analyzed patients with PD can be considered as a pilot study. The very good consistency of the bradykinesia and short-step parameters, which is described in the pilot study of Kluge et al., can be confirmed in this study with a larger sample size consisting of 37 probands. The Bland–Altman diagram showed a similar bias of a stride length of 2.95 cm and gait velocity of 2.46 cm/s (Kluge et al.: stride length: 1.4 cm; gait velocity: 1.2 cm/s). 

### 4.2. Shuffling Gait Parameters

For shuffling gait parameters, significant differences between both systems were identified in this study. The validation of shuffling gait parameters as well as the rel. stance and rel. swing phase between MGL and MC has not been identified as a central theme in the literature until now. The Bland–Altman plots presented an increasing average difference of relative stance and swing phase with increasing duration of relative stance time and synchronic decreasing duration of relative swing time. Additionally, differences of the average values of HS and TO are increasing with larger foot angles. A different gait phase determination such as first floor contact or heel-strike moment of both gait analysis systems may be a possible explanation. In the MGL, the algorithm defines the gait phases, whereas in the MC the investigator identifies the gait phases by using the video image as reference [13,19]. Further studies need to clarify the impact of HS and TO definition on MGL and MC to evaluate if one of those gait analysis system may define gait phases more precisely. The comparison of TC between MGL and a MC is already represented in the literature [21]. As described in this paper, we defined the TC with the MC as the maximal foot height during the swing phase relative to the height at flat foot in the stance phase of the toe marker. The Bland–Altman diagram showed a constant bias for TC of 2.25 ± 1.01 cm in our study and, therefore, a similar result to Kanzler et al. (TC = 1.69 ± 0.70 cm). The analyzed cohort in Kanzler et al. included 744 strides of 20 probands, which is similar to the present. Compared with our study, they investigated healthy subjects and did not include PD patients. Taking into account the presented ICC results, it becomes apparent that bradykinesia and short-step gait parameters in patients with PD can be reliably compared between MGL and MC. However, rel. stance and swing time, as well as shuffling gait parameters, are only comparable with limited extent between both gait analysis systems.

### 4.3. Clinical Relevance of Sensor-Based Gait Parameters

To investigate if the MGL detects clinically relevant gait impairment, the data of patients with PD were additionally compared with those of a healthy age-matched control group. The aim was to evaluate if MGL and MC similarly identify Parkinsonian gait and differentiate from those of a control group. The results showed that PD patients walked with a significantly reduced gait speed and stride length, and stride time increased by around 11% compared to healthy controls. Additionally, the heel-strike angle and TC were reduced. Similar results were previously described in Schlachetzki et al. where they measured a reduced gait speed and stride length of 190 patients with PD compared with a healthy control group (*n* = 101). Stride time of the patients was increased by ~5%, HS of the first floor contact was reduced by ~22%, and TC was limited [12]. Despite a larger study cohort in the study of Schlachetzki et al., the results of this study demonstrate that both MGL and MC can comparably differentiate patients with PD from a healthy control group. In addition, Schlachetzki et al. pointed out that TC differs significantly from a control group independently from the patient’s disease stage [12]. To evaluate if we can also define one gait parameter that already differed in an early PD stage from a healthy control group, we separated the patients by their disease stages and compared the gait results with the control group. In contrast to the already published results after significance-level correction, we could not identify one gait parameter that differed in early stages from a healthy control group. Additionally, Pistacchi et al. realized in early Parkinson’s disease stages (H&Y 2 and 2.5) a significantly reduced gait speed and cadence of 44 patients compared with a healthy control group [26]. Post hoc analysis in this study showed that all gait parameters, except rel. stance and rel. swing phase, were significantly distinguished between patients with H&Y 3 and the control group with both gait analysis systems comparable. Due to a similar sample size, only the results of Pistacchi et al. are directly comparable with those in our study. Initiating and deceleration strides, as well as turning angles, were not included in our study. The focus of this study was the comparison between MGL and MC; therefore, the measurement volume was restricted to the middle part of the 10 m gait bout. As Nguyen et al. presented, gait initiation, termination and transitioning predicted motor impairment in patients with PD better than the straight strides did [27]. This may be one explanation that both systems could not detect gait parameter differences between patients staged with H&Y 1 + 2 and controls. Taking this into account, the results already published in the literature could not be confirmed in this study completely. Since patients with H&Y 4 use a walking aid, this should be considered for correct interpretation of sensor signals. Sensor-based and motion-capture gait parameters were distinguished from a healthy control group solely in an advanced Parkinson’s disease stage (H&Y 3). Gait speed, stride length, HS and TC showed with r ≥ 0.50 the highest effect size thereby. 

### 4.4. Use Cases for Gold Standard and Mobile Systems

The technical validation of a mobile sensor-based gait analysis system with MC for patients with PD is the basis for the use of this mobile system in a clinically relevant context. Motion capture systems are still considered as the gold standard to analyze the patient’s gait and provide highly accurate results over short-distance standardized walking tests in the hospital [6,7]. However, a gait analysis with motion capture systems is still very time consuming, expensive and absolutely limited to the lab environment [4]. In contrast, mobile systems have the potential to record gait patterns over several hours in flexible environments, including a patient’s daily life. This is a major benefit for physicians since clinical diagnostics is mostly limited to a short timeframe in the hospital during the doctor’s visit. Furthermore, wearable sensors complement clinical scores, as they analyze gait fluctuations that appear especially in PD patients during the day [8,9,10,28]. In future studies, it should be investigated if wearable sensors may be used in other orthopedic, neuromuscular and neurological diseases to evaluate disease evolution, compliance to therapies and indication for surgeries.

### 4.5. Limitations

One limitation of the study is the camera setup, which limits the measurement volume of the MC to three left and three right steps of the patients. As solely the middle part of the gait cycle could be compared between the gait analysis systems, gait variability parameters were not included in this study. Straight strides in the measurement volume do not completely reflect daily life scenarios. The synchronization was realized by video image. Although a stride-by-stride synchronization was conducted, the results must be interpreted cautiously, since the overlay with the help of the video image was implemented by the investigator and could not be fully automated. The different sample sizes of the patient group (PD) compared with the healthy control group may influence the results, as well as the diverse distribution of the H&Y stages.

## 5. Conclusions

The results of this study demonstrate that sensor-based gait speed, stride length and stride time, as well as TC, were accurately captured in comparison with a motion capture system. These results indicate that robust sensor-based gait parameters were generated by MGL. Furthermore, both systems similarly measure a significant difference between patients with PD and a healthy age-matched control group. Compared with previous large cohort studies, we did not identify a parameter that differed between the control group and early PD stages. The limitation of the measurement volume due to the camera setup may be one explanation. In summary, the results show that sensor-based bradykinesia and short-step parameters are highly comparable with gait parameters of an MC. These findings indicate that MGL can be used for gait pattern monitoring in flexible environments, including recordings over several hours in patient’s everyday life. Future studies need to investigate whether sensor-based gait parameters objectively reflect therapy response and disease progression in PD.

## Figures and Tables

**Table 1:** Characteristics of PD patients and control group. H&Y: Hoehn and Yahr disease stage; MMST: Minimal Mental Status; *p* < 0.05 Mann–Whitney U test; ° chi-squared test.

	Validation Group PD (*n* = 37)	Control Group (*n* = 13)	*p*
**Age** (years)	70.8 ± 9.3	72.2 ± 28.8	0.575
**Gender** (m:f)	20:17	8:6	0.843 °
**UPDRS-III** (score)	19.9 ± 9.0	na	na
**H&Y** (score)	3.1 ± 0.7	na	na
1; 1.5	1	na	na
2; 2.5	5	na	na
3; 3.5	21	na	na
4	12	na	na
**MMST** (score)	28.4 ± 1.8	na	na
**Gait** (without:with wheeled walker)	25:12	14:0	0.046 °

**Table 2:** Inter-class correlation coefficient (ICC); maximum and minimum of 95% confidence interval.

	ICC	95% Confidence Interval		*p*	α_c_ *
		Minimum	Maximum		
**Bradykinesia/short steps**					
gait speed	0.986	0.975	0.992	**<0.001**	<0.001
stride length	0.985	0.974	0.992	**<0.001**	<0.001
stride time	0.966	0.942	0.981	**<0.001**	<0.001
rel. stance time	0.964	0.938	0.980	**<0.001**	<0.001
rel. swing time	0.964	0.938	0.980	**<0.001**	<0.001
**Shuffling gait**					
HS	0.822	0.162	0.943	**<0.001**	<0.001
TO	0.903	0.505	0.967	**<0.001**	<0.001
TC	0.900	0.425	0.967	**<0.001**	<0.001

* Significance level corrected by Benjamini–Hochberg False Rate Discovery (α_c_) with *p* < 0.05; significant results are highlighted in **bold**.

**Table 3:** Gait parameters recorded with MGL and MC (mean, standard deviation (std) and significance level (*p*)).

	System	Group Comparison			
		Mean	std	*p*	α_c_ *
**Bradykinesia/short steps**					
gait speed [m/s]	MGL	0.98	0.32	0.780	0.780
	MC	0.98	0.32		
stride length [m]	MGL	1.14	0.29	0.122	0.195
	MC	1.13	0.28		
stride time [s]	MGL	1.20	0.16	**0.003**	0.006
	MC	1.19	0.16		
stance time [%]	MGL	65.40	2.47	0.192	0.219
	MC	65.14	2.68		
swing time [%]	MGL	34.60	2.47	0.192	0.219
	MC	34.86	2.68		
**Shuffling gait**					
HS [°]	MGL	8.97	5.52	**<0.001**	<0.001
	MC	16.49	7.00		
TO [°]	MGL	61.27	11.84	**<0.001**	<0.001
	MC	52.95	9.01		
TC [cm]	MGL	5.87	2.77	**<0.001**	<0.001
	MC	8.32	2.99		

* Significance level corrected by Benjamini–Hochberg False Rate Discovery (α_c_) with *p* < 0.05; significant results are highlighted in **bold.**

**Table 4:** Group comparison of MGL and MC and effect size (r) compared between patients subdivided in H&Y stage 2, 3 and 4 (II, III, IV) and control group (c).

		Group Comparison			Effect Size	*p* (Benjamini–Hochberg Post Hoc Test)					
		*p*	α_c_	z	r	c–II	α_c_ * c–II	C–III	α_c_ * c–III	c–IV	α_c_ * c–IV
**Bradykinesia/short steps**											
gait speed [m/s]	MGL	**0.002**	0.003	3.784	0.53	0.172	0.356	**0.007**	0.011	**<0.001**	<0.001
	MC	**0.001**	0.002	3.880	0.54	0.151	0.243	**0.005**	0.010	**<0.001**	<0.001
stride length [m]	MGL	**0.001**	0.003	3.917	0.55	0.262	0.356	**0.023**	0.031	<0.001 *	<0.001
	MC	**0.001**	0.002	3.926	0.55	0.247	0.247	**0.024**	0.024	**<0.001**	<0.001
stride time [s]	MGL	**0.025**	0.002	−2.798	0.39	0.267	0.356	**0.005**	0.001	**0.005**	0.005
	MC	**0.008**	0.008	−2.958	0.41	0.201	0.247	**0.003**	0.001	**0.003**	0.003
stance time [%]	MGL	**0.044**	0.044	−2.832	0.40	0.464	0.464	0.159	0.159	**0.005**	0.005
	MC	**0.002**	0.027	−3.766	0.53	0.029	0.116	**0.020**	0.023	**<0.001**	<0.001
swing time [%]	MGL	**0.044**	0.044	2.832	0.40	0.464	0.464	0.159	0.159	**0.005**	0.005
	MC	**0.002**	0.027	3.766	0.53	0.029	0.116	**0.020**	0.023	**<0.001**	<0.001
**Shuffling gait**											
HS [°]	MGL	**0.001**	0.003	3.740	0.52	0.011	0.088	**0.001**	0.004	**<0.001**	<0.001
	MC	**0.001**	0.002	3.856	0.54	0.060	0.158	**0.003**	0.010	**<0.001**	<0.001
TO [°]	MGL	**0.002**	0.003	3.701	0.52	0.204	0.356	**0.004**	0.010	**<0.001**	<0.001
	MC	**0.003**	0.003	3.463	0.48	0.217	0.247	**0.005**	0.010	**0.001**	0.001
TC [cm]	MGL	**<0.001**	<0.001	4.370	0.61	0.024	0.096	**0.001**	0.004	**<0.001**	<0.001
	MC	**0.001**	0.002	4.209	0.69	0.079	0.158	**0.008**	0.013	**<0.001**	<0.001

* Significance level corrected by Benjamini–Hochberg False Rate Discovery (α_c_) with *p* < 0.05; significant results are highlighted in **bold.**

## Data Availability

Date will be made available on request to the correspondent author’s email with appropriate justification.
